# Desulfosporosinus paludis sp. nov., an acidotolerant sulphate-reducing bacterium isolated from moderately acidic fen soil

**DOI:** 10.1099/ijsem.0.006648

**Published:** 2025-01-27

**Authors:** Stefan Dyksma, Meina Neumann-Schaal, Mathias Müsken, Michael Pester

**Affiliations:** 1 Department of Microorganisms, Leibniz Institute DSMZ – German Collection of Microorganisms and Cell Cultures, Braunschweig, Germany; 2 Chemical Analytics and Metabolomics, Leibniz Institute DSMZ – German Collection of Microorganisms and Cell Cultures, Braunschweig, Germany; 3 Braunschweig Integrated Centre of Systems Biology (BRICS), Braunschweig, Germany; 4 Central Facility for Microscopy, Helmholtz Centre for Infection Research, Inhoffenstraße 7, Braunschweig, Germany; 5 Institute of Microbiology, Technical University of Braunschweig, Braunschweig, Germany

**Keywords:** acidophile, fens, freshwater wetlands, sulphate reduction, sulphur cycle

## Abstract

An obligately anaerobic, spore-forming sulphate-reducing bacterium, strain SB140^T^, was isolated from a long-term continuous enrichment culture that was inoculated with peat soil from an acidic fen. Cells were immotile, slightly curved rods that stained Gram-negative. The optimum temperature for growth was 28 °C. Strain SB140^T^ grew at pH 4.0–7.5 with an optimum pH of 6.0–7.0 using various electron donors and electron acceptors. Yeast extract, sugars, alcohols and organic acids were used as electron donors for sulphate reduction. SB140^T^ additionally used elemental sulphur and nitrate as electron acceptors but not sulphite, thiosulphate or iron(III) provided as ferrihydrite and fumarate. The 16S rRNA gene sequence placed strain SB140^T^ in the genus *Desulfosporosinus* of the phylum *Bacillota*. The predominant cellular fatty acids were iso-C_15 : 0_ (52.6%) and 5,7 C_15 : 2_ (19.9%). The draft genome of SB140^T^ (5.42 Mbp in size) shared 77.4% average nucleotide identity with the closest cultured relatives *Desulfosporosinus acididurans* M1^T^ and *Desulfosporosinus acidiphilus* SJ4^T^. On the basis of phenotypic, phylogenetic and genomic characteristics, SB140^T^ was identified as a novel species within the genus *Desulfosporosinus*, for which we propose the name *Desulfosporosinus paludis* sp. nov. The type strain is SB140^T^ (=DSM 117342^T^=JCM 39521^T^).

## Introduction

Sulphate reduction is a globally important process that drives organic matter mineralization in anoxic environments [1]. The metabolic potential for sulphate reduction has been encountered in representatives across 19 bacterial and 2 archaeal phyla [2]. Most sulphate-reducing bacteria (SRB) are dependent on low-molecular-weight fermentation products that are derived from the breakdown of organic polymers [1]. Nevertheless, many SRB have the metabolic flexibility to utilize alternative electron acceptors, disproportionate sulphur compounds, ferment and interact in syntrophic associations [1,3]. SRB can provide important ecosystem services in low-sulphate environments such as freshwater wetlands by their potential to attenuate methane emissions due to direct competition with methanogenic *Archaea* [4]. Members of the genus *Desulfosporosinus* are widespread in freshwater wetlands worldwide [5] and have been identified as potential keystone species in peat soil from an acidic fen where they substantially contributed to overall sulphate reduction and carbon mineralization activity [6]. Three representatives of the genus *Desulfosporosinus* were so far isolated from acidic environments and validly described: *Desulfosporosinus acidiphilus* isolated from acidic mine drainage [7], *Desulfosporosinus acididurans* isolated from acidic river sediments [8] and *Desulfosporosinus metallidurans* from a microbial mat of an acidic puddle at a gold mining site [9]. In the present study, we characterize a novel acidotolerant *Desulfosporosinus* bacterium, isolated from a moderately acidic (pH 4–5) fen.

## Enrichment and isolation

Strain SB140^T^ was isolated from a continuously operated bioreactor [10] that was inoculated with peat soil from an acidic fen located in the Fichtel Mountains, Bavaria, Germany (50°08′38″N, 11°51′41″E). Enrichment and isolation were carried out exclusively in a liquid medium. The initial enrichment was set up with the medium used for bioreactor operation, which was composed of KH_2_PO_4_ 0.1 g l^−1^, (NH_4_)_2_SO_4_ 0.066 g l^−1^, MgSO_4_×7 H_2_O 0.123 g l^−1^, CaCl_2_×2 H_2_O 0.02 g l^−1^, glucose monohydrate 0.1 g l^−1^, pectin 0.5 g l^−1^, trace element solution according to medium DSM141 [11] (Leibniz Institute DSMZ-German Collection of Microorganisms and Cell Cultures) 1 ml l^−1^, 20× vitamin solution according to DSM141 0.5 ml l^−1^ and MES hydrate 1.95 g l^−1^ [10] using the bioreactor content as inoculum (1%). To specifically enrich spore-forming bacteria, a fraction (1 ml) from the initial sulphate-reducing enrichment culture was pasteurized (80 °C, 20 min) and subsequently transferred to anoxic medium DSM1250 supplemented with KH_2_PO4 (0.1 g l^−1^) and fructose (5 mM) without reducing agents. Phosphate buffer in the medium was replaced by MES hydrate (1.95 g l^−1^), and the pH was adjusted to 5.5. Strain SB140^T^ was isolated using dilution-to-extinction cultivation at 20 °C.

## Phylogeny and genomic analysis

**Fig. 1.**
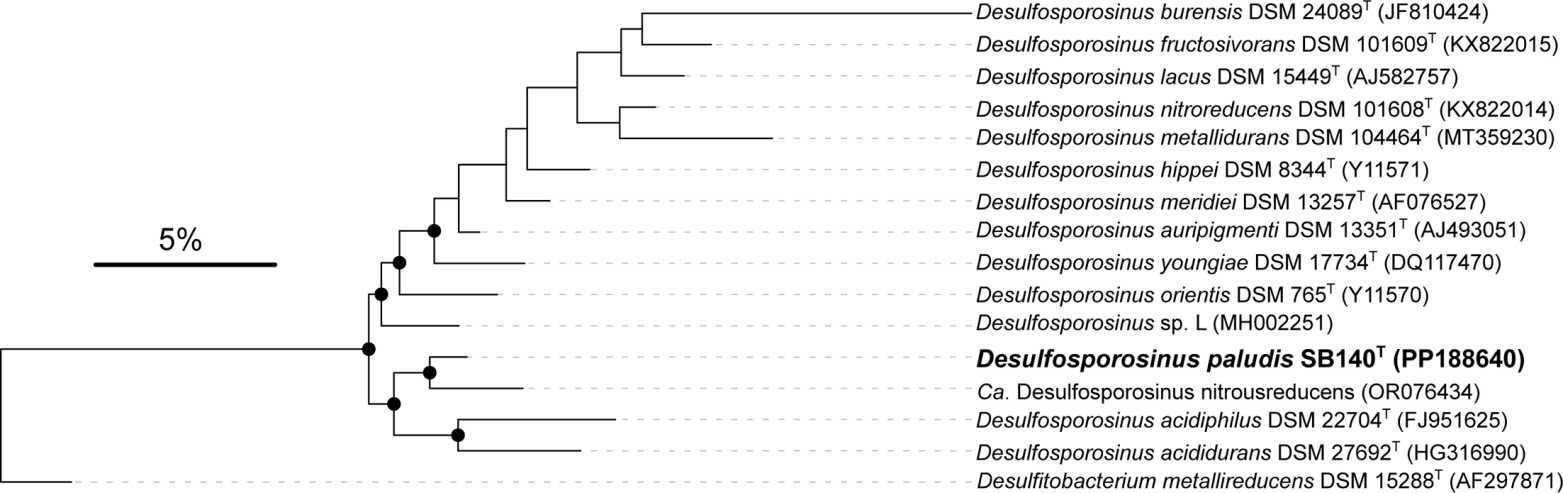
Phylogenetic tree based on 16S rRNA gene sequences of strain SB140^T^ and related species of the genus *Desulfosporosinus. Desulfitobacterium metallireducens* DSM 15288^T^ (AF297871) was used as an outgroup. Black dots denote bootstrap support ≥90%. The scale bar represents 5% sequence divergence.

**Fig. 2.**
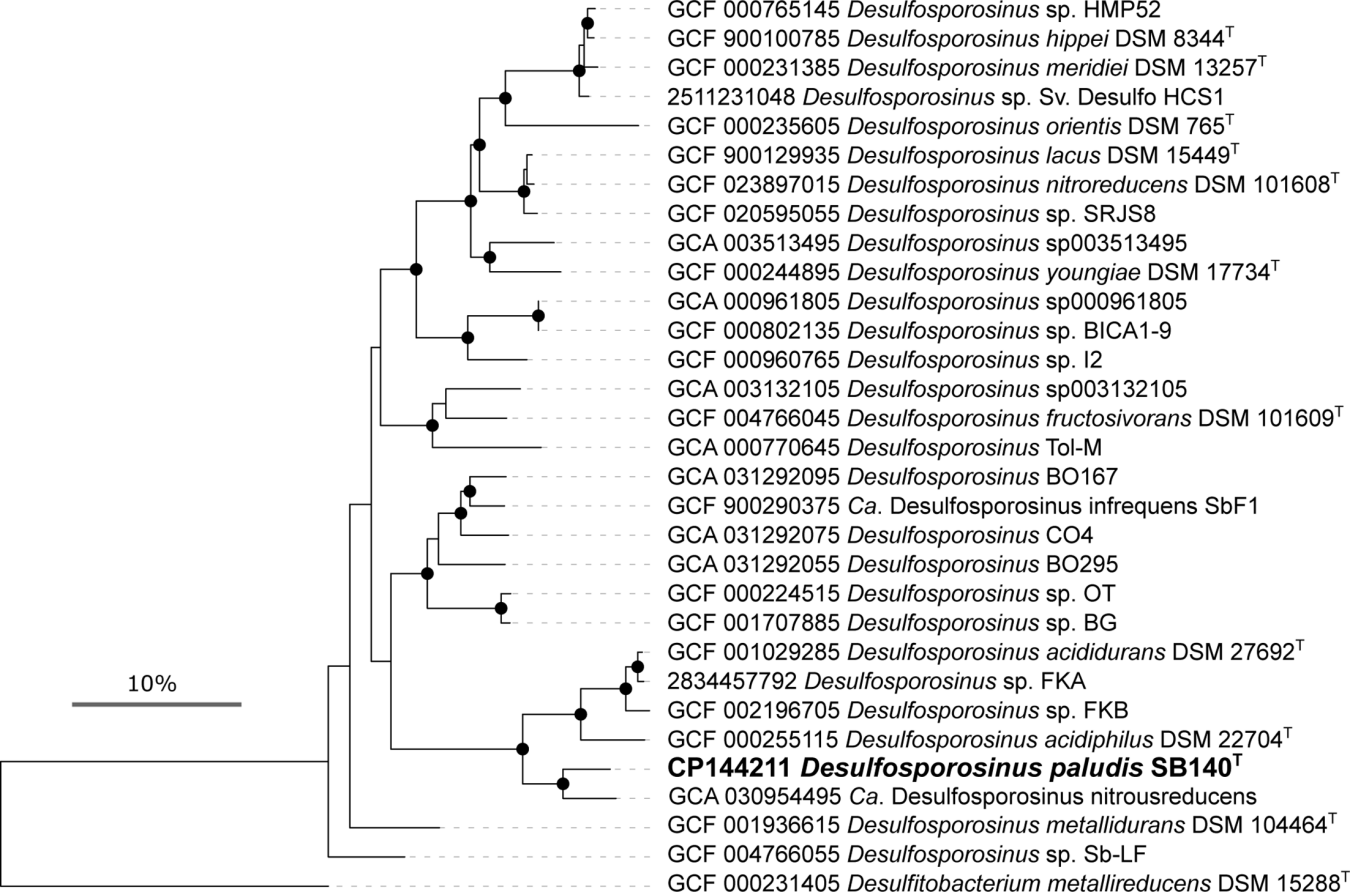
Phylogenomic reconstruction of the genus *Desulfosporosinus* based on the concatenated alignment of 120 single-copy bacterial marker proteins. *Desulfitobacterium metallireducens* DSM 15288^T^ (GCF 000231405) was used as an outgroup. Black dots denote bootstrap support ≥90%. The scale bar represents 10% sequence divergence.

DNA was extracted using the AllPrep PowerViral DNA/RNA kit (Qiagen, Netherlands) according to the manufacturer’s instructions. The Ligation Sequencing Kit SQK-LSK114 (Oxford Nanopore Technologies, Oxford, UK) was used for library preparation. Sequencing was performed on a MinION with a Flongle flow cell (FLO-FLG114, Oxford Nanopore Technologies, Oxford, UK), assembled with Flye (version 2.9.2) and annotated with MetaERG [12] and RAST [13]. Similar to other *Desulfosporosinus* species [9,14], strain SB140^T^ had multiple rRNA operons. Nine gene copies each that encode the 5S, 16S and 23S rRNA genes were identified in its genome. Sequence similarities of its 16S rRNA genes were 99.4–100.0%. The sequence with the highest number of identical copies found in the genome (*n*=4) was used as a representative for the reconstruction of the 16S rRNA gene phylogeny (Fig. 1). The 16S rRNA gene and genome sequence of strain SB140^T^ were deposited in GenBank under the accession numbers PP188640 and CP144211, respectively. Sequences were aligned with MAFFT (E-INS-i) [15], and tree calculation was performed with IQ-TREE 2 (version 2.2.0.3) using ultrafast bootstraps (*n*=1000) after automatic substitution model selection (TPM3+I+I+R4) [16]. Phylogenetic reconstruction placed strain SB140^T^ within the genus *Desulfosporosinus* with *D. acididurans* (96.7% 16S rRNA gene identity) and *D. acidiphilus* (96.0% identity) as close relatives (Fig. 1). To confirm the phylogenetic placement of strain SB140^T^, a phylogenomic tree was constructed based on 120 single-copy marker genes extracted and aligned with GTDBtk [17]. Tree calculation was performed with IQ-TREE 2 using ultrafast bootstraps (*n*=1000) after automatic substitution model selection (Q.plant+F+R4). Strain SB140^T^ formed a monophyletic cluster with *D. acididurans* and *D. acidiphilus*, as well as with some metagenome-assembled genomes that have been recovered from acidic environments (Fig. 2). Digital DNA–DNA hybridization values were highest between SB140^T^ and *D. acidiphilus* (23.2%, formula *d_4_*) as revealed by using the TYGS platform [18]. The highest genome-wide average nucleotide identity (ANI) [19] was shared with *Candidatus* (*Ca*.) *Desulfosporosinus nitroreducens* (84.5%), an N_2_O-reducing *Desulfosporosinus* sp. described in an acidic co-culture [20]. ANI values to *Desulfosporosinus* strains previously described from the same fen, such as the isolate *Desulfosporosinus* sp. Sb-LF [21] or the metagenome-assembled genome of *Ca*. *Desulfosporosinus* infrequens SbF1A [22], were only 75.0 and 74.6%, respectively. The ANI values between SB140^T^ and the two validly described acidophilic representatives *D. acididurans* and *D. acidiphilus* were 77.4% and the strain shared 73.2% ANI with the type species *Desulfosporosinus orientis*. In addition, average amino acid identity values (Fig. S1, available in the online Supplementary Material) were all above the threshold for genus delineation within the family *Desulfitobacteria* (65.3%) [23], which confirmed that strain SB140^T^ belongs to the genus *Desulfosporosinus*.

The draft genome size of strain SB140^T^ was 5 424 553 bp on a single contig with a G+C content of 42.26 mol%, which is comparable to other *Desulfosporosinus* species [14]. A total of 66 tRNAs and 5132 protein-coding genes were predicted with MetaERG. All genes of the Wood–Ljungdahl pathway (WLP) were identified in the genome of SB140^T^, similar to other genome-sequenced *Desulfosporosinus* species [14,22, 24,26]. This pathway exerts multiple functions in anaerobic bacteria, such as providing an electron sink for redox balancing in acetogens growing on various substrates [27], allowing syntrophic and SRB to utilize acetate as a carbon and energy source [28,29] or being an important carbon fixation pathway [30]. An NiFe group 1 a hydrogenase for hydrogen oxidation was also encoded in the genome, indicating that the WLP may be used for autotrophic growth. The genome further contained all genes necessary for the utilization of, e.g. formate, butyrate, pyruvate and lactate, as well as all genes of the complete canonical sulphate reduction pathway (*sat; aprAB-qmoAB; dsrABCDMKJOP*). The genomic potential for nitrous oxide reduction (*nosZ* gene) in strain SB140^T^ was shared by *Ca*. *D. nitroreducens* and few other validly described *Desulfosporosinus* species (*D. nitroreducens*, *D. youngiae* and *D. meridiei*), suggesting a potentially important function as scavengers of the greenhouse gas N_2_O in anoxic environments.

## Phenotypic characterization and physiology

**Fig. 3.**
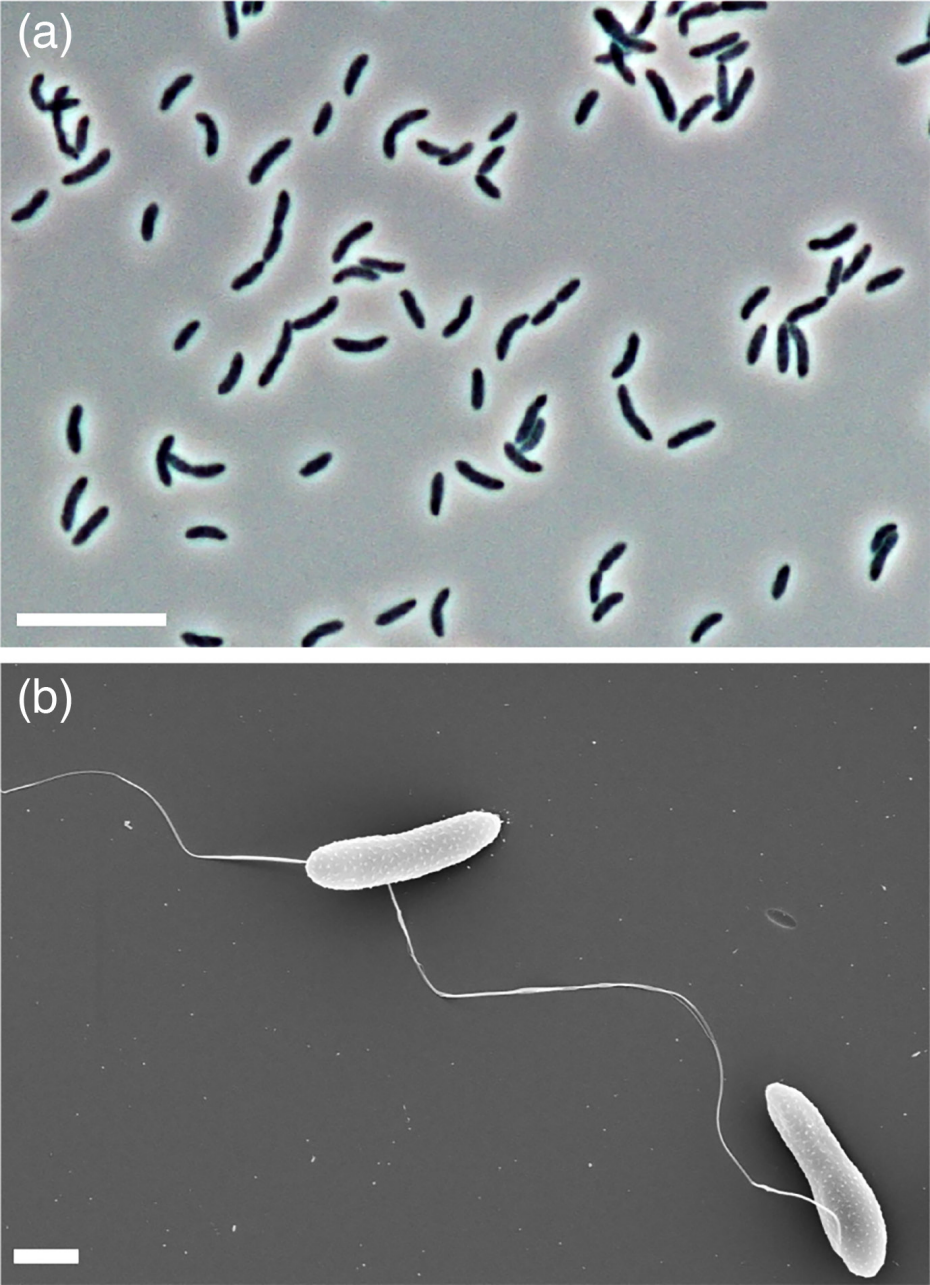
Phase-contrast (**a**) and scanning electron microscopy image (**b**) of strain SB140^T^ cultivated for 6 days at 28 °C with fumarate and sulphate. The scale bars represent 10 and 1 µm, respectively.

Morphology and motility were observed using phase-contrast microscopy and scanning electron microscopy as described recently [31]. Cells appeared as slightly curved rods, 2.9–5.8 µm long and 0.7–1.4 µm wide (Fig. 3). Subterminal endospores were occasionally observed. Gram-stain was performed according to a standard protocol [32]. Cells stained Gram-negative, similar to other representatives of the genus *Desulfosporosinus* [7,9]. Subpolar flagella were observed (Fig. 3), although the cells were not motile.

Growth experiments to test for temperature, pH and NaCl optimum were performed in triplicates in 25 ml culture tubes with the modified medium DSM1250 used for isolation, supplemented with glucose (5 mM) as substrate. In all growth experiments, growth was determined by following OD (measured at 600 nm) over time. Growth was tested in the range 4–37 °C. The optimum temperature (28 °C) was used to test growth at different pH (3.0–8.5) and NaCl concentrations (0–3% w:v). Strain SB140^T^ grew between 0 and 1% NaCl and between pH 4.0 and 7.5 (Table 1). The optimum pH for growth (pH 6–7) revealed that strain SB140^T^ was acidotolerant [33], unlike its closest acidophilic relatives, *D. acididurans* and * D. acidiphilus*. No growth was observed at pH 3.0 and pH 8.5. The following electron donors were tested in duplicate cultures with sulphate as an electron acceptor: H_2_, lactate, pyruvate, malate, citrate, fumarate, succinate, benzoate, formate, acetate, propionate, butyrate, glucose, fructose, xylose, methanol, ethanol, propanol, glycerol and yeast extract. In addition to OD_600_ measurements, sulphide production was determined using the methylene blue method [34]. Strain SB140^T^ grew on a wide range of organic substrates (Table 1) with sulphate as an electron acceptor. Propionate did not support the growth of the strain, consistent with the lack of methylmalonyl-CoA pathway genes for propionate oxidation in its genome. Moreover, no growth was observed with formate, acetate, malate, citrate and benzoate. Autotrophic growth with H_2_/CO_2_ without acetate was possible. In the medium to test for alternative electron acceptors and fermentation, the amount of MgSO_4_×7 H_2_O was reduced to 0.01 g l^−1^ and (NH_4_)_2_SO_4_ was replaced by 0.1 g l^−1^ NH_4_Cl. Strain SB140^T^ fermented lactate, pyruvate, fumarate, fructose and yeast extract. Weak fermentative growth was also observed with glucose, methanol and glycerol. Ethanol was not fermented but served as an electron donor for sulphate reduction. Ethanol was therefore used as a substrate to test growth with the following alternative electron acceptors: elemental sulphur, thiosulphate, sulphite, nitrate, fumarate and Fe(III) (provided as ferrihydrite). Strain SB140^T^ did not use sulphite, thiosulphate and Fe(III) as electron acceptors. The products of fermentation and sulphate respiration were analysed using ion chromatography as follows. Anions were separated in a SykroGel AX300 column (Sykam, Germany) using 5 mM NaCO_3_ as an eluent. Organic acids were separated in a SykroGel EX450 column (Sykam, Germany) using 7% acetonitrile and 0.7 mM perfluorobutanoic acid as eluent. Both anions and organic acids were quantified using a conductivity detector. Substrates were incompletely oxidized to acetate and acetate was also a major fermentation product. Microaerobic growth with 1% O_2_ in the headspace was not possible.

**Table 1.** Comparison of the major characteristics that differentiate strain SB140^T^ (1) from its closest phylogenetic relatives *D. acididurans* M1^T^ (2), *D. acidiphilus* SJ4^T^ (3) and *D. metallidurans* OL^T^ (4) and *D. orientis* Singapore I^T^, representing the type species of the genus Data were obtained from Sánchez-Andrea *et al*. [8] for strain M1^T^, from Alazard *et al*. [7] for strain SJ4^T^ unless otherwise indicated and from Panova *et al*. [9] for strain OL^T^. Data for strain Singapore I^T^ were obtained from Klemps *et al*. [39], Stackebrandt *et al*. [40], Robertson *et al*. [41] and Ramamoorthy *et al*. [42].

Characteristics	1	2∗	3†	4‡	5§
Type strain	SB140^T^	M1^T^	SJ4^T^	OL^T^	Singapore I^T^
Isolation source	Acidic fen	Acidic river sediment	Acid mine drainage sediment	Acidic puddle at gold mine tailings	Soil
Cell size (µm)	2.9–5.8×0.7–1.4	3–5×0.7	4–7×0.8–1.0	2–3×0.50–0.53	3–5×0.7–1,0¶
Endospore position	Subterminal	Subterminal	Subterminal	Subterminal	Subterminal to terminal
Motility	−	Variable	−	−	+
Temperature range, optimum (°C)	20–28, 28	15–40, 30	25–40, 30	4–37, 28	30–42, 30–37
pH range, optimum	4.0–7.5, 6–7	3.8–7, 5.5	3.6–5.6, 5.2	4–7, 5.5	5.6–7.4, 6.4–7.0
NaCl range, optimum (%)	0–1, 0.1	0–1.5, 0.6	0–0.6	0–6, 0–0.1	0–<5∗∗
Major respiratory quinones	MK-7	MK-7	nd	nd	MK-7¶
Electron donors with sulphate					
H_2_/CO_2_	+	+	+	+	+
Lactate	+	+	(+)	+	+
Pyruvate	+	+	+∗	+	+¶,∗∗, ††
Malate	−	+	−	+	−∗∗
Citrate	−	−	−	−	−∗
Succinate	+	−	−	−	−∗, ††
Fumarate	+	+	−	−	+∗∗, ††
Benzoate	−	−	−	nd	−∗,∗∗, ††
Formate	−	+	−	+	+
Acetate	−	−	−	(+)	∗,∗∗, ††
Propionate	−	−	−	+	−∗∗, ††
Butyrate	+	+	−	−	+††
Glucose	+	+	(+)	+	-††
Fructose	+	+	+	+	-¶,∗∗
Xylose	+	+	−	nd	−∗
Methanol	+	(+)	−	nd	+∗∗, ††
Ethanol	+	+	−	+	+∗∗, ††
1-Propanol	+	+	−	−	+
2-Propanol	+	nd	nd	nd	nd
Glycerol	+	+	+	+	−††, +∗
Yeast extract	+	+	-, +∗	nd	+††
Electron acceptors with glycerol∗,†,§, lactate† or ethanol‡‡					
Sulphate	+	+	+	+	+
Elemental sulphur	+	+	-, +∗	−	+∗∗, ††
Thiosulphate	−	+	+∗	+	+∗^,^¶^,^∗∗, ††
Sulphite	−	−	−	+	+∗∗,††
Fumarate	−	−	−	+	−, +
Fe(III)	−	+	−	−	+∗,∗∗, ††
Nitrate	+	+	(+)∗	+	−∗∗
Fermentation in the absence of sulphate					
Lactate	+	−	−	+	+
Pyruvate	+	+	−	+	−, (+)∗
Fumarate	+	+	(+)∗	nd	+∗
Formate	−	−	−	nd	+∗
Glucose	(+)	−	nd	+	−, +∗
Fructose	+	nd	−	nd	nd
Methanol	(+)	−	−	nd	+¶
Ethanol	−	−	−	nd	−∗, +¶
Glycerol	(+)	−	-∗	nd	+∗
Yeast extract	+	+	-∗	nd	nd

nd, not determined; +, growth; (+), weak growth; −, no growth.

*Data from Sánchez-Andrea *et al*., 2015.

†Data from Alazard *et al*., 2010 unless otherwise indicated.

‡Data from Panova *et al*., 2021.

§Data from Klemps *et al*., 1985.

¶Data from Stackebrandt *et al.*, 1997.

∗∗Data from Robertson *et al*., 2001.

††Data from Ramamoorthy *et al*., 2006.

‡‡This study.

## Chemotaxonomy

For chemotaxonomic comparison, strain SB140^T^ and *D. acididurans* M1^T^ were grown in the modified, MES-buffered medium DSM1250 with glycerol as a substrate. Respiratory quinones were purified from wet biomass obtained from cultures in the late exponential growth phase and analysed by HPLC as described earlier [35]. Similar to *D. acididurans* [8], the major menaquinone of strain SB140^T^ was MK-7 (>99%) with a minor presence of MK-8. Cellular fatty acids were extracted, methylated and analysed as fatty acid methyl esters using a combined approach of gas chromatography with flame ionization detection (GC-FID) for quantification and gas chromatography with mass spectrometry (GC-MS) for identification, followed by specific derivatization methods for the determination of double bond positions [35,36]. The dominant fatty acids that constitute more than 5% of the total fatty acids of strain SB140^T^ were C_15 : 0_ (5.5%), iso-C_15 : 0_ (52.6%) and 5,7 C_15 : 2_ (19.9%) (Table 2). For the 5,7 C_15 : 2_ fatty acid, the data indicate that one of the double bonds is not in *cis* isomerism as expected in conjugated dienoic fatty acids, but this could not be further resolved due to the lack of authentic standards of the isomers. A detailed cellular fatty acid profile is provided as Supplementary material.

**Table 2.** Cellular fatty acid composition of strain SB140^T^ and its closest phylogenetic relatives *D. acididurans* M1^T^ and *D. acidiphilus* SJ4^T^ in comparison to the acidophilic *D. metallidurans* OL^T^ and *D. orientis* Singapore I^T^, representing the type species of the genus Numbers depicted in the table are percentages of total fatty acids. Major cellular fatty acids that constitute more than 5% of the total fatty acids in at least one strain are shown. Major cellular fatty acids (>5%) of individual strains are indicated in bold. Please note that different methodological approaches were used to determine cellular fatty acids. The strains SB140^T^, M1^T^, SJ4^T^ and Singapore I^T^ were grown with glycerol and sulphate. Strain OL^T^ was grown with lactate and sulphate. A detailed fatty acid profile of strain SB140^T^ and the closest relative strain M1^T^, as determined in this study, is provided in Table S1.

Fatty acid	SB140^T^	M1^T^	SJ4^T^	OL^T^	Singapore I^T^
C_14 : 0_	2.3	3.6	**15.7**	**7.5**	2.6
C_15 : 0_	**5.5**	nd	nd	–	nd
iso-C_15 : 0_	**52.6**	**31.1**	**28.1**	–	–
5,7 C_15 : 2_	**19.9**	–	2.8	nd	1.7
C_16 : 1_ *cis*9	1.2	2.1	3.6	**39.0**	**5.9**
C_16 : 0_	0.6	**10.7**	**18.7**	**12.1**	**43.8**
C_16 : 0_ DMA	0.2	**9.4**	**10.8**	**8.1**	**6.3**
C_17 : 0_ cyc	–	**12.1**	4.2	1.4	0.3
iso-C_17 : 1_ *cis*9	**5.9**	nd	nd	nd	nd
C_18 : 1_ *cis*11 DMA	–	1.6	–	nd	**17.1**
C_18 : 1_ *cis*11	–	0.7	–	2.5	**12.5**

–, not detected; nd, not determined or not reported.

Strain M1T and Singapore IT, data from Sánchez-Andrea *et al*., 2015.

Strain SJ4T, data from Alazard *et al*., 2010.

Strain OLT, data from Panova *et al*., 2021.

## Conclusion

The phylogenetic, physiological and chemotaxonomic results showed that the new isolate SB140^T^ represents a new species within the genus *Desulfosporosinus*. Representatives of the genus *Desulfosporosinus* form a monophyletic clade within the family *Desulfitobacteriaceae* and are distinct from representatives of the related genus *Desulfitobacterium* by their ability to perform sulphate reduction. *Desulfosporosinus* spp. are further characterized by their ability to grow chemoautrotrophically on CO_2_ and H_2_, their incomplete oxidation of organic substrates to acetate and CO_2_ and by representing strictly anaerobic, rod-shaped, mesophilic, endospore-forming bacteria. All *Desulfosporosinus* spp. possess MK-7 as the main respiratory quinone [8,9, 37, 38]. Strain SB140^T^ fulfils all these criteria to be placed into the genus *Desulfosporosinus*. Strain SB140^T^ differs from all other described *Desulfosporosinus* spp. by its inability to utilize thiosulphate. Furthermore, it differs from its closest phylogenetic relatives *D. acididurans* M1^T^ and *D. acidiphilus* SJ4^T^ by ANI values of 77.4% and the utilization of succinate with sulphate as an electron acceptor. This was further supported by the fatty acid pattern of strain SB140^T^, which contained 5,7 C_15 : 2_ as the second most dominant fatty acid (19.9%) in comparison to its close relatives (<2.8%), while it contained only negligible quantities of C_16 : 0_ (0.6%) and C_16 : 0_ DMA (0.2%) in comparison to its closest relatives (>8%) (Table 2).

## Description of *Desulfosporosinus paludis* sp. nov.

*Desulfosporosinus paludis* sp. nov. (pa.lu′dis. L. gen. n. *paludis*, of a swamp, of a marsh, of a bog).

Cells are immotile, slightly curved rods, 0.7–1.4 µm in width and 2.9–5.8 µm in length. Subterminal endospores are produced occasionally. The temperature range for growth is 20–28 °C, with an optimum at 28 °C. No growth occurred at 10 °C and 37 °C. Neutrophilic, with an optimum growth at pH 6–7. Growth occurs at pH 4.0–7.5, but not at pH 3 and pH 8.5. The upper limit for salt tolerance is 1% (w:v). Sulphate reduction is observed with H_2_, lactate, pyruvate, fumarate, succinate, butyrate, glucose, fructose, xylose, methanol, ethanol, propanol, glycerol and yeast extract as electron donors. Sulphate, elemental sulphur and nitrate are used as electron acceptors. Fermentation of lactate, pyruvate, fumarate, fructose and yeast extract is positive. The following substrates are not used as electron donors: formate, acetate, propionate, malate, citrate and benzoate. Menaquinone MK-7 is the major respiratory quinone and the dominant cellular fatty acids are iso-C_15 : 0_ (52.6%) and 5,7 C_15 : 2_ (19.9%).

The type strain SB140^T^ (=DSM 117342^T^=JCM 39521^T^) was isolated from a continuous enrichment culture that was inoculated with peat soil from an acidic fen (Fichtel Mountains, Bavaria, Germany). Based on the genome sequence, the G+C content of the type strain is 42.3 mol%. The GenBank accession numbers for the 16S rRNA gene and the genome sequence of strain SB140^T^ are PP188640 and CP144211, respectively.

## supplementary material

10.1099/ijsem.0.006648 Uncited Supplementary Material 1.

## Abbreviations

AAI: average amino acid identity
ANI: average nucleotide identity
dDDH: digital DNA–DNA hybridization
SRB: sulphate-reducing bacteria
