# Supplementary material for: Desulfosporosinus paludis sp. nov., an acidotolerant sulphate-reducing bacterium isolated from moderately acidic fen soil
Source: Int J Syst Evol Microbiol. 2025 Jan 27;75(1):006648. doi: 10.1099/ijsem.0.006648 (PMC11771766; doi:10.1099/ijsem.0.006648)
Supplement: Uncited Supplementary Material 1. [file ijsem-75-06648-s001.pdf]

Supplementary Material

***Desulfosporosinus paludis* sp. nov., an acidotolerant sulfate-reducing bacterium isolated from moderately acidic fen soil.**

Stefan Dyksma*[,1], Meina Neumann-Schaal[2,3], Mathias Müsken[4] and Michael Pester[1,3,5]

1 - Leibniz Institute DSMZ – German Collection of Microorganisms and Cell Cultures, Department of Microorganisms, Braunschweig, Germany

2 - Leibniz Institute DSMZ – German Collection of Microorganisms and Cell Cultures, Chemical Analytics and Metabolomics, Braunschweig, Germany

3 - Braunschweig Integrated Centre of Systems Biology (BRICS), Rebenring 56, D-38106 Braunschweig

4 - Central Facility for Microscopy, Helmholtz Centre for Infection Research, Inhoffenstraße 7, 38124 Braunschweig, Germany

5 - Technical University of Braunschweig, Institute of Microbiology, Braunschweig, Germany

* To whom correspondence should be addressed: Leibniz Institute DSMZ – German Collection of Microorganisms and Cell Cultures, Department of Microorganisms, Braunschweig, Germany

Tel +49 531 2616420, stefan.dyksma@dsmz.de

	Desulfosporosinus acidiphilus (GCF_000255115)	*Desulfosporosinus youngiae* (GCF_000244895)	*Desulfosporosinus orientis* (GCF_000235605)	*Desulfosporosinus meridiei* (GCF_000231385)	*Desulfosporosinus* sp. Sv. Desulfo HCS1 (2511231048)	Desulfosporosinus sp. OT (GCF_000224515)	*Desulfosporosinus lacus* (GCF_900129935)	*Desulfosporosinus* sp. Tol-M (GCA_000770645)	*Desulfosporosinus* sp. HMP52 (GCF_000765145)	*Desulfosporosinus* sp. I2 (GCF_000960765)	*Desulfosporosinus acididurans* (GCF_001029285)	*Desulfosporosinus hippei* (GCF_900100785)	*Desulfosporosinus* sp. BG (GCF_001707885)	*Desulfosporosinus* sp. FKB (GCF_002196705)	*Desulfosporosinus* sp. FKA (2834457792)	*Desulfosporosinus fructosivorans* (GCF_004766045)	*Desulfosporosinus* sp. Sb-LF (GCF_004766055)	***Desulfosporosinus paludis* strain SB140 (CP144211)**	*Desulfosporosinus* sp. sp003132105 (GCA_003132105)	*Desulfosporosinus* sp. sp003513495 (GCA_003513495)	*Ca*. Desulfosporosinus nitrousreducens (GCA_030954495)	*Desulfosporosinus* sp. BICA1-9 (GCF_000802135)	*Desulfosporosinus* sp. sp000961805 (GCA_000961805)	*Desulfosporosinus metallidurans* (GCF_001936615)	*Desulfosporosinus* sp. SRJS8 (GCF_020595055)	*Desulfosporosinus nitroreducens* (GCF_023897015)	*Ca*. Desulfosporosinus infrequens SbF1 (GCF_900290375)	*Desulfosporosinus* sp. MAG BO167 (GCA_031292095)	*Desulfosporosinus* sp. MAG BO295 (GCA_031292055)	*Desulfosporosinus* sp. MAG CO4 (GCA_031292075)	*Desulfitobacterium metallireducens* (GCF_000231405)
Desulfosporosinus acidiphilus (GCF_000255115)	100	68.5	69.6	69.5	68.7	70.7	69.5	66.4	69.5	70.6	82.3	68.9	71.5	82.2	82.0	70.0	71.3	78.0	69.1	68.6	76.3	70.1	69.8	71.6	69.0	69.3	69.4	68.9	70.1	69.9	61.7
Desulfosporosinus youngiae (GCF_000244895)	68.5	100	76.9	79.0	77.8	72.6	79.4	69.0	78.5	74.7	68.3	78.4	73.1	68.9	68.1	73.8	74.2	70.4	69.8	81.3	69.9	74.2	73.8	73.2	79.1	79.5	69.8	70.1	70.7	70.5	61.6
Desulfosporosinus orientis (GCF_000235605)	69.6	76.9	100	78.0	77.6	72.6	77.9	67.0	78.0	74.1	69.0	77.5	72.8	69.7	68.7	73.1	72.8	70.7	69.5	76.8	69.9	73.9	73.3	72.1	78.0	78.4	69.0	69.3	70.2	69.7	61.2
Desulfosporosinus meridiei (GCF_000231385)	69.5	79.0	78.0	100	92.9	73.5	81.5	68.3	93.6	74.9	69.1	94.8	73.5	69.5	69.1	74.3	74.0	70.7	70.1	78.7	69.9	74.6	74.2	72.9	81.4	81.5	69.7	69.7	71.1	70.3	62.0
Desulfosporosinus sp. Sv. Desulfo HCS1 (2511231048)	68.7	77.8	77.6	92.9	100	72.5	81.0	67.1	92.9	74.7	68.6	93.1	72.8	69.0	68.8	73.6	73.8	70.3	69.5	79.0	69.5	75.1	74.6	72.8	80.7	81.7	68.9	69.3	70.0	69.7	61.3
Desulfosporosinus sp. OT (GCF_000224515)	70.7	72.6	72.6	73.5	72.5	100	74.3	70.2	73.4	75.6	70.7	73.5	93.6	70.7	70.2	75.9	76.7	73.2	73.1	72.5	71.2	75.2	74.3	77.3	74.4	75.0	77.3	77.6	79.0	77.6	61.3
Desulfosporosinus lacus (GCF_900129935)	69.5	79.4	77.9	81.5	81.0	74.3	100	69.7	81.9	78.1	69.8	81.5	75.7	69.8	69.4	76.8	75.3	71.9	72.2	81.0	70.2	77.8	77.1	74.7	92.5	93.8	71.6	72.0	72.6	71.9	62.1
Desulfosporosinus sp. Tol-M (GCA_000770645)	66.4	69.0	67.0	68.3	67.1	70.2	69.7	100	68.0	69.9	66.2	67.8	71.5	65.7	65.6	73.0	70.2	67.5	72.1	68.3	66.6	70.2	69.8	71.1	69.3	69.6	69.2	69.4	69.3	69.5	59.5
Desulfosporosinus sp. HMP52 (GCF_000765145)	69.5	78.5	78.0	93.6	92.9	73.4	81.9	68.0	100	75.0	69.1	94.2	73.1	69.1	68.8	73.9	74.4	70.8	70.3	80.6	69.4	75.1	74.7	73.3	81.0	81.6	70.0	69.7	70.5	70.4	61.7
Desulfosporosinus sp. I2 (GCF_000960765)	70.6	74.7	74.1	74.9	74.7	75.6	78.1	69.9	75.0	100	69.7	75.1	76.5	69.8	69.7	77.0	75.5	72.8	73.7	76.0	70.9	82.8	82.2	78.8	76.7	78.1	72.3	72.9	73.4	72.0	62.0
Desulfosporosinus acididurans (GCF_001029285)	82.3	68.3	69.0	69.1	68.6	70.7	69.8	66.2	69.1	69.7	100	69.0	71.5	91.1	95.9	69.1	71.1	77.1	68.3	68.1	76.0	68.6	68.3	71.1	69.2	69.6	68.9	69.0	69.7	69.9	61.3
Desulfosporosinus hippei (GCF_900100785)	68.9	78.4	77.5	94.8	93.1	73.5	81.5	67.8	94.2	75.1	69.0	100	73.4	69.1	68.9	73.9	74.1	70.9	69.8	78.8	70.0	75.1	74.5	72.9	81.4	81.5	69.8	69.6	70.9	70.1	61.6
Desulfosporosinus sp. BG (GCF_001707885)	71.5	73.1	72.8	73.5	72.8	93.6	75.7	71.5	73.1	76.5	71.5	73.4	100	71.2	70.8	76.3	77.4	74.4	74.5	73.5	72.1	75.3	74.5	78.0	74.4	75.5	78.3	78.5	79.6	79.1	61.7
Desulfosporosinus sp. FKB (GCF_002196705)	82.2	68.9	69.7	69.5	69.0	70.7	69.8	65.7	69.1	69.8	91.1	69.1	71.2	100	91.1	69.5	71.6	77.4	68.8	68.2	76.6	68.3	68.2	72.2	69.6	69.7	69.3	69.2	70.5	69.8	61.2
Desulfosporosinus sp. FKA (2834457792)	82.0	68.1	68.7	69.1	68.8	70.2	69.4	65.6	68.8	69.7	95.9	68.9	70.8	91.1	100	68.8	70.5	76.7	68.1	67.8	75.4	68.3	68.1	71.5	69.2	69.5	68.5	68.7	69.6	69.1	61.1
Desulfosporosinus fructosivorans (GCF_004766045)	70.0	73.8	73.1	74.3	73.6	75.9	76.8	73.0	73.9	77.0	69.1	73.9	76.3	69.5	68.8	100	75.6	72.4	77.4	74.2	70.3	77.3	76.3	76.4	76.4	77.2	73.8	74.6	74.8	74.3	61.8
Desulfosporosinus sp. Sb-LF (GCF_004766055)	71.3	74.2	72.8	74.0	73.8	76.7	75.3	70.2	74.4	75.5	71.1	74.1	77.4	71.6	70.5	75.6	100	74.9	75.2	74.3	72.4	75.0	74.7	79.2	75.1	75.9	74.4	74.7	75.3	74.9	62.7
***Desulfosporosinus paludis* strain SB140 (CP144211)**	78.0	70.4	70.7	70.7	70.3	73.2	71.9	67.5	70.8	72.8	77.1	70.9	74.4	77.4	76.7	72.4	74.9	100	71.1	70.1	84.6	71.3	71.3	74.6	71.0	71.6	72.2	71.5	72.4	72.2	62.1
Desulfosporosinus sp. sp003132105 (GCA_003132105)	69.1	69.8	69.5	70.1	69.5	73.1	72.2	72.1	70.3	73.7	68.3	69.8	74.5	68.8	68.1	77.4	75.2	71.1	100	70.9	69.7	73.1	72.4	76.4	71.2	72.1	73.0	73.1	73.0	72.3	61.3
Desulfosporosinus sp. sp003513495 (GCA_003513495)	68.6	81.3	76.8	78.7	79.0	72.5	81.0	68.3	80.6	76.0	68.1	78.8	73.5	68.2	67.8	74.2	74.3	70.1	70.9	100	69.4	78.0	77.6	73.7	79.9	81.6	69.4	69.7	70.0	70.4	61.1
Ca. Desulfosporosinus nitrousreducens (GCA_030954495)	76.3	69.9	69.9	69.9	69.5	71.2	70.2	66.6	69.4	70.9	76.0	70.0	72.1	76.6	75.4	70.3	72.4	84.6	69.7	69.4	100	69.7	69.5	72.7	70.0	70.2	70.5	69.5	71.4	71.1	61.6
Desulfosporosinus sp. BICA1-9 (GCF_000802135)	70.1	74.2	73.9	74.6	75.1	75.2	77.8	70.2	75.1	82.8	68.6	75.1	75.3	68.3	68.3	77.3	75.0	71.3	73.1	78.0	69.7	100	99.9	76.8	76.9	78.6	71.7	71.6	72.8	72.0	61.6
Desulfosporosinus sp. sp000961805 (GCA_000961805)	69.8	73.8	73.3	74.2	74.6	74.3	77.1	69.8	74.7	82.2	68.3	74.5	74.5	68.2	68.1	76.3	74.7	71.3	72.4	77.6	69.5	99.9	100	76.1	76.1	77.9	71.1	71.1	72.0	71.4	61.2
Desulfosporosinus metallidurans (GCF_001936615)	71.6	73.2	72.1	72.9	72.8	77.3	74.7	71.1	73.3	78.8	71.1	72.9	78.0	72.2	71.5	76.4	79.2	74.6	76.4	73.7	72.7	76.8	76.1	100	74.0	75.0	74.8	75.0	75.8	75.0	62.6
Desulfosporosinus sp. SRJS8 (GCF_020595055)	69.0	79.1	78.0	81.4	80.7	74.4	92.5	69.3	81.0	76.7	69.2	81.4	74.4	69.6	69.2	76.4	75.1	71.0	71.2	79.9	70.0	76.9	76.1	74.0	100	92.0	71.0	71.3	72.4	71.3	61.5
*Desulfosporosinus nitroreducen*s (GCF_023897015)	69.3	79.5	78.4	81.5	81.7	75.0	93.8	69.6	81.6	78.1	69.6	81.5	75.5	69.7	69.5	77.2	75.9	71.6	72.1	81.6	70.2	78.6	77.9	75.0	92.0	100	71.0	71.7	72.3	71.5	62.0
Ca. Desulfosporosinus infrequens SbF1 (GCF_900290375)	69.4	69.8	69.0	69.7	68.9	77.3	71.6	69.2	70.0	72.3	68.9	69.8	78.3	69.3	68.5	73.8	74.4	72.2	73.0	69.4	70.5	71.7	71.1	74.8	71.0	71.0	100	84.8	81.1	83.7	60.8
Desulfosporosinus sp. MAG BO167 (GCA_031292095)	68.9	70.1	69.3	69.7	69.3	77.6	72.0	69.4	69.7	72.9	69.0	69.6	78.5	69.2	68.7	74.6	74.7	71.5	73.1	69.7	69.5	71.6	71.1	75.0	71.3	71.7	84.8	100	80.0	83.9	60.4
Desulfosporosinus sp. MAG BO295 (GCA_031292055)	70.1	70.7	70.2	71.1	70.0	79.0	72.6	69.3	70.5	73.4	69.7	70.9	79.6	70.5	69.6	74.8	75.3	72.4	73.0	70.0	71.4	72.8	72.0	75.8	72.4	72.3	81.1	80.0	100	79.5	60.7
Desulfosporosinus sp. MAG CO4 (GCA_031292075)	69.9	70.5	69.7	70.3	69.7	77.6	71.9	69.5	70.4	72.0	69.9	70.1	79.1	69.8	69.1	74.3	74.9	72.2	72.3	70.4	71.1	72.0	71.4	75.0	71.3	71.5	83.7	83.9	79.5	100	60.6
Desulfitobacterium metallireducens (GCF_000231405)	61.7	61.6	61.2	62.0	61.3	61.3	62.1	59.5	61.7	62.0	61.3	61.6	61.7	61.2	61.1	61.8	62.7	62.1	61.3	61.1	61.6	61.6	61.2	62.6	61.5	62.0	60.8	60.4	60.7	60.6	100

Supplementary Figure S1. Average amino acid identity matrix of 30 *Desulfosporosinus* genomes and metagenome assembled genomes.

Supplementary Table S1. Detailed cellular fatty acid profiles of strain SB140^T and the close phylogenic relative *D. acididurans* M1^T grown with glycerol as determined in this study. Numbers depicted in the table are percentages of total fatty acids.

**Fatty acid**	**Strain SB140^T**	***D. acididurans* M1^T**
C14:0 ISO	<0.1	0.1
C14:0	2.3	2.0
C15:1 ISO CIS 7	5.0	2.9
C14:0 DMA	-	0.2
C15:0 ISO	52.6	35.5
C15:0 ANTEISO	2.5	0.2
C15:0	5.5	2.0
C15:0 ISO DMA	<0.1	2.4
C15:2 5,7 (one or two double bond positions in trans)	19.9	-
C15:0 DMA	<0.1	0.4
C16:0 ISO	0.4	0.5
C16:1 CIS 7	0.2	0.6
C16:1 CIS 9	1.2	1.9
C16:0	0.6	8.5
C16:0 ISO DMA	-	0.2
C16:1 CIS 7 DMA	-	0.2
C16:1 CIS 9 DMA	-	2.2
C17:1 ISO CIS 9	5.9	9.4
C16:0 DMA	0.2	4.6
C17:0 ISO	2.1	5.1
C17:0 ANTEISO	0.4	0.2
C17:2 (positions not determined)	0.2	0.3
C17:1 ISO DMA (position not determined)	-	7.7
C17:1 ISO DMA (position not determined)	-	0.6
C17:0	0.1	0.3
C17:0 ISO DMA	0.1	6.6
C17:0 ANTEISO DMA	-	0.1
C17:1 DMA (position not determined)	-	<0.1
C17:1 DMA (position not determined)	-	1.0
C17:0 DMA	-	0.6
C18:1 CIS 9	-	0.4
C18:1 CIS 11	-	0.4
C18:0	0.7	1.4
C18:1 CIS 9 DMA	-	0.6
C18:1 CIS 11 DMA	-	0.7
C18:0 DMA	-	0.1

-, not detected
